# Fatty acid composition of ground-beef products and their plant-based meat substitutes available in Hungary

**DOI:** 10.3389/fnut.2026.1732327

**Published:** 2026-02-25

**Authors:** Viktor Koczka, Tamás Marosvölgyi, Zoltán Szabó, Timea Dergez, Éva Szabó

**Affiliations:** 1 Doctoral School of Health Sciences, Faculty of Health Sciences, University of Pécs, Pécs, Hungary; 2 Department of Biochemistry and Medical Chemistry, Medical School, University of Pécs, Pécs, Hungary; 3 Institute of Bioanalysis, Medical School, University of Pécs, Pécs, Hungary; 4 Institute of Nutritional Sciences and Dietetics, Faculty of Health Sciences, University of Pécs, Pécs, Hungary

**Keywords:** fatty acid, lipid content, nutritional index, plant-based diet, plant-based meat alternatives, polyunsaturated fatty acid, vegan products

## Abstract

**Background:**

In recent years, plant-based diets have gained popularity. The food industry has responded by introducing a range of alternative products that significantly differ from whole-food, plant-based diets in terms of their composition and processing levels. This study aimed to compare the fatty acid composition and fatty acid-based nutritional quality indices of ground beef-based foods with those of their plant-based counterparts available in the Hungarian market.

**Methods:**

This study examined six plant-based and four beef hamburger patties, along with one plant-based and one beef minced meat product, each with three distinct expiration dates. Following homogenization and lipid extraction, the fatty acid composition was analyzed by gas chromatography. Based on the fatty acid values, several nutritional indices were calculated, including the unsaturation index (UI), atherogenicity index, thrombogenicity index, and hypocholesterolemic/hypercholesterolemic index (hHI).

**Results:**

Significant differences (*p* < 0.01) in fat content were observed between plant-based and animal-based products, based on both label information and gravimetric measurements (plant-based: 10.25% [8.60%; 14.87%], animal-based: 19.67% [16.16%; 26.68%], median [Q1; Q3]). Distinct fatty acid composition profiles were identified between and within the product groups for both animal- and plant-based products. Except for one product, plant-based alternatives exhibited higher UI and hHI (UI: 129.62 [96.84; 146.10]; hHI: 50.13 [45.69; 54.14]) than beef-based products (UI: 8.18 [3.13; 11.59]; hHI: 1.35 [1.23; 1.43]).

**Conclusion:**

The findings indicate that plant-based meat alternatives (except those containing coconut oil) have lower saturated and higher polyunsaturated fatty acid compositions than beef-based products, leading to more beneficial nutritional value. Further analytical and clinical studies are necessary to provide a more comprehensive understanding of the long-term health effects of these foods.

## 1 Introduction

Meat consumption has been essential to human evolution, serving as a vital source of macro- and micronutrients (1). The essential nutrients found in meat, including high-quality protein, heme iron, essential fatty acids, vitamin B12, other B vitamins, retinol, zinc, and bioactive compounds such as creatine and carnosine, may have contributed to the development of the large human brain and supported cognitive functioning and physical growth (2). Over the past two decades, the global population has grown, and rapid economic development has driven meat demand up by nearly 60%, surpassing 360 million tons, with further growth anticipated. Large quantities of beef, pork, and poultry are consumed worldwide. Industrial meat production significantly contributes to greenhouse gas emissions, deforestation, and biodiversity loss, making it a critical factor in sustainability (3–5).

Excessive meat consumption poses risks to both the environment and human health. The World Health Organization’s International Agency for Research on Cancer (IARC) has classified processed meat as carcinogenic and red meat as probably carcinogenic, primarily because of the evidence linking them to colorectal cancer (6). Furthermore, the consumption of red and processed meat may adversely affect the incidence of cardiovascular diseases (7), type 2 diabetes (8), and various other cancers, including esophageal, gastric, lung, and breast cancer (9–12).

Recent studies have highlighted the protective effects of substituting animal protein sources with plant-based sources against certain diseases and mortalities (13–16). The demand for plant-based meat substitutes (PBMS) is increasing as consumers seek healthier, more sustainable, and ethical alternatives (17, 18). These products offer solutions to the challenges associated with meat production and the adverse health effects of excessive meat consumption (19).

The fatty acid (FA) composition of foods varies and plays a crucial role in nutrition. A high intake of certain saturated fatty acids (SFA) is associated with elevated low-density lipoprotein (LDL) cholesterol levels and an increased risk of cardiovascular disease (20–22). Conversely, the consumption of monounsaturated and polyunsaturated fatty acids (MUFA and PUFA, respectively) is associated with improved blood lipid profiles and a reduced risk of cardiovascular disease and metabolic disorders (23, 24). Consequently, dietary guidelines recommend limiting SFA (and eliminating *trans* fats) while emphasizing the importance of adequate PUFA consumption for cardiovascular health (25, 26). Among PUFAs, essential fatty acids (EFAs) cannot be synthesized by the human body and must be obtained from the diet. Both omega-6 (n-6) essential linoleic acid (C18:2n-6, LA) and omega-3 (n-3) essential alpha-linolenic acid (C18:3n-3, ALA), along with their most important longer-chain derivatives, arachidonic acid (C20:4n-6, AA), eicosapentaenoic acid (C20:5n-3, EPA), and docosahexaenoic acid (C22:6n-3, DHA), have demonstrated beneficial health effects that can influence various aspects of human physiology (27–30).

PBMSs contain various vegetable oils and fats that replicate the juiciness and flavor of meat products. Common fat ingredients include coconut, rapeseed, sunflower, soybean, and palm oil. The choice of fat source determines the FA profile of the final product, which can vary significantly. When coconut or palm oil is used, the composition contains a higher proportion of SFAs, whereas in other cases, MUFAs and PUFAs predominate in different proportions (31–33). According to the NOVA classification (34), most PBMS, although not all, are considered ultra-processed foods, similar to meat-based burger patties, raising questions about their health impacts (35, 36).

To date, few studies have examined the composition of plant-based meat substitutes using analytical techniques. While some authors have focused on mineral (31, 37–39), protein (31, 37), or fatty acid content (40, 41), others have determined the major macro- and micronutrients (38).

Given the increasing prevalence of PBMSs in Hungary and the limited availability of analytical studies on this topic, this study aimed to investigate the differences in fat content and FA composition between minced beef-based products (BBPs) and PBMSs. Understanding the nutritional differences between these substitutes and traditional meat products is crucial. Therefore, we also aimed to compare the fatty acid-derived nutritional indices of these products.

## 2 Materials and methods

### 2.1 Sample collection

The selection criteria targeted chilled (non-frozen), semi-finished products of both plant and animal origins, including all locally available products in the selected stores. Between August and October 2023, seven plant-based and five animal-based products were purchased from leading food retail chains in Pécs, Hungary. The included products were hamburger patties, but there was also one minced product from each origin. Three separate samples of each product with different expiration dates were collected to account for the potential batch variability. All products were purchased in vacuum-packed form, transported to the laboratory in a cooler bag, and stored at 5 °C until further processing before their expiration date. Homogenization was performed using an electric meat grinder by running each sample through the machine twice. The grinder was thoroughly cleaned between samples to prevent cross-contamination. The homogenized samples were stored at −80 °C until chemical analysis.

### 2.2 Reagents and standards

The reagents used in this study have been previously described (33). Fatty acid methyl esters (FAMEs) were analyzed by calculating the area under the curve (AUC). Fatty acids were identified based on the retention times of external standards, including the 37 Component FAME Mix (Supelco Merck KGaA, Darmstadt, Germany), The Bacterial Acid Methyl Esters CP Mixture (Matreya LLC., State College, PA, USA), and GLC-463, −473, and −674 (Nu-Check-Prep, Elysian, MN, USA). Peaks were identified by comparing them with authentic mixtures of the weighed FAME methyl ester, and the individual FA response factors, determined from these weighed standards, along with the percentage AUC (relative concentration; w/w%), were used to calculate the weight percentage of each determined FA (42).

### 2.3 Lipid extraction and fatty acid analysis

We measured 100 mg of pre-homogenized material using a precision analytical balance (OHAUS Explorer EX225D/AD) and placed it in a specialized tissue grinder (KIMBLE Dounce). The material was further crushed and transferred into a pre-weighed screw-cap test tube, with 2.5 mL of chloroform added in several portions. Subsequently, 2.5 mL of methanol was introduced into the test tube and incubated at 37 °C for 20 min. Subsequently, 1.25 mL of water was added, and the mixture was incubated in a refrigerator for 20 min. The test tubes were then centrifuged at 3000 RPM for 15 min at 4 °C (Sanyo-Harrier 18/80, refrigerated). The lower chloroform phase, which contained the lipids, was extracted using a Pasteur pipette and transferred to a pre-weighed tube for further analysis. Chloroform was removed using nitrogen in an automatic evaporator (Biotage TurboVAP). The tube containing the dry, evaporated lipids was weighed to a constant weight on an analytical balance, and the lipid content of the sample was calculated.

We added 5 mL of chloroform to the tubes containing a known amount of lipids that had been evaporated to dryness. After homogenization, we measured a solution with 1 mg of lipids in a screw-top test tube and evaporated the chloroform. The steps for converting lipids to methyl esters have been described previously (33). The fatty acid composition was determined using a Thermo Trace 1,300 gas chromatograph (GC) equipped with an autosampler AI1310, flame ionization detector (FID), and programmed temperature vaporization (PTV) injector. Separation was achieved using a capillary column (Agilent J&W VF-23 ms, 60 m × 0.25 mm × 0.25 μm; Agilent J&W Scientific, Folsom, CA, USA). The column oven temperature was initially set at 120 °C for 2 min, then increased to 250 °C at a rate of 3 °C·min^−1^, and held at 250 °C for 3 min. The carrier gas was H_2_ at 1.8 mL/min. Fatty acids were determined in the range of C6:0 to C26:0. The fatty acid composition of each sample was determined based on 12 chromatograms (two parallel runs of the samples after two independent analytical procedures from samples collected on three different dates). Chromatograms were evaluated using Chromeleon 7.1 software (version 7.1, Thermo Fisher Scientific, Sunnyvale, CA, USA).

### 2.4 Nutritional index calculation

The nutritional indices of fatty acids were calculated based on the formulas published by Chen et al. (43) and the Healthy Fatty Index (HFI) by Dal Bosco et al. (44), as follows:

Essential fatty acids (EFA):


EFA=C18:2n−6+C18:3n−3

n–3/n–6 polyunsaturated fatty acid (PUFA) ratio:


$$\frac{\mathrm{\Sigma n}-3\;\text{PUFA}}{\mathrm{\Sigma n}-6\;\text{PUFA}}$$

Σn-3 PUFA denotes the sum of n–3 polyunsaturated fatty acids (C18:3n-3 + C20:5n-3 + C22:5n-3 + C22:6n-3).

Σn-6 PUFA denotes the sum of n–6 polyunsaturated fatty acids (C18:2n-6 + C18:3n-6 + C20:2n-6 + C20:3n-6 + C20:4n-6 + C22:2n-6 + C22:4n-6).

Unsaturation index (UI):

UI = 1 × (% monoenoics) + 2 × (% dienoics) + 3 × (% trienoics) + 4 × (% tetraenoics) + 5 × (% pentaenoics) + 6 × (% hexaenoics).

monoenoics: C14:1n-5 + C15:1n-5 + C16:1n-7 + C17:1n-7 + C18:1n-12 + C18:1n-9 + C18:1n-7 + C20:1n-12 + C20:1n-9 + C22:1n-9; dienoics: C18:2n-6 + C20:2n-6 + C22:2n-6; trienoics: C18:3n-3 + C18:3n-6 + C20:3n-6; tetraenoics: C20:4n-6 + C22:4n-6; pentaenoics: C20:5n-3 + C22:5n-3; hexaenoics: C22:6n-3.

Atherogenicity index (AI):


$$\mathrm{AI}=\frac{C12:0+4\times C14:0+C16:0}{\text{ΣUFA}}$$

ΣUFA denotes the sum of unsaturated fatty acids (C14:1n-5 + C15:1n-5 + C16:1n-7 + C17:1n-7 + C18:1n-12 + C18:1n-9 + C18:1n-7 + C18:2n-6 + C18:3n-3 + C18:3n-6 + C20:1n-12 + C20:1n-9 + C20:2n-6 + C20:3n-6 + C20:4n-6 + C20:5n-3 + C22:1n-9 + C22:2n-6 + C22:4n-6 + C22:5n-3 + C22:6n-3).

Thrombogenicity index (TI)


$$\mathrm{TI}=\frac{C14:0+C16:0+C18:0}{0.5\times \text{ΣMUFA}+0.5\times \mathrm{\Sigma n}-6\;\text{PUFA}+3\times \mathrm{\Sigma n}-3\;\text{PUFA}+\frac{\mathrm{\Sigma n}-3\;\text{PUFA}}{\mathrm{\Sigma n}-6\;\text{PUFA}}}$$

ΣMUFA denotes the sum of monounsaturated fatty acids (C14:1n-5 + C15:1n-5 + C16:1n-7 + C17:1n-7 + C18:1n-12 + C18:1n-9 + C18:1n-7 + C20:1n-12 + C20:1n-9 + C22:1n-9).

Σn-6 PUFA denotes the sum of n–6 polyunsaturated fatty acids (C18:2n-6 + C18:3n-6 + C20:2n-6 + C20:3n-6 + C20:4n-6 + C22:2n-6 + C22:4n-6).

Σn-3 PUFA denotes the sum of n–3 polyunsaturated fatty acids (C18:3n-3 + C20:5n-3 + C22:5n-3 + C22:6n-3).

Hypocholesterolemic/hypercholesterolemic index (hHI)


$$\mathrm{hHI}=\frac{\mathrm{cis}\;C18:1n-9+\text{ΣPUFA}}{C12:0+C14:0+C16:0}$$

ΣPUFA denotes the sum of polyunsaturated fatty acids (C18:2n-6 + C18:3n-6 + C18:3n-3 + C20:2n-6 + C20:3n-6 + C20:4n-6 + C20:5n-3 + C22:2n-6 + C22:4n-6 + C22:5n-3 + C22:6n-3).

Healthy fatty index (HFI)


$$\mathrm{HFI}=\frac{(2\times \text{ΣMUFA})+(4\times \mathrm{\Sigma n}-6\;\text{PUFA})+(8\times \mathrm{\Sigma n}-3\;\text{PUFA})+\frac{\mathrm{\Sigma n}-3\;\text{PUFA}}{\mathrm{\Sigma n}-6\;\text{PUFA}}}{(1\times \text{ΣSAT})+(0.5\times \text{ΣMUFA})+(0.25\times \mathrm{\Sigma n}-6\;\text{PUFA})+(0.125\times \mathrm{\Sigma n}-3\;\text{PUFA})+\frac{\mathrm{\Sigma n}-6\;\text{PUFA}}{\mathrm{\Sigma n}-3\;\text{PUFA}}}$$

ΣMUFA denotes the sum of monounsaturated fatty acids (C14:1n-5 + C15:1n-5 + C16:1n-7 + C17:1n-7 + C18:1n-12 + C18:1n-9 + C18:1n-7 + C20:1n-12 + C20:1n-9 + C22:1n-9).

Σn-6 PUFA denotes the sum of n–6 polyunsaturated fatty acids (C18:2n-6 + C18:3n-6 + C20:2n-6 + C20:3n-6 + C20:4n-6 + C22:2n-6 + C22:4n-6).

Σn-3 PUFA denotes the sum of n–3 polyunsaturated fatty acids (C18:3n-3 + C20:5n-3 + C22:5n-3 + C22:6n-3).

ΣSAT denotes the sum of all determined saturated fatty acids (C6:0 - C26:0, excluding C7:0 and C22:0).

### 2.5 Statistical analysis

Statistical significance was set at *p* < 0.05. Data were analyzed using the Kruskal-Wallis and Mann–Whitney tests, with Bonferroni correction applied for multiple comparisons. All statistical analyses were performed using IBM SPSS Statistics for Windows, version 29.0 (SPSS Inc., Chicago, IL, USA). Fatty acid composition is expressed as the weight percentage (w/w%) of total fatty acids. Data are presented as medians with interquartile ranges (IQR) in the tables and figures.

## 3 Results

### 3.1 Label information

We analyzed the nutritional information presented on product labels, with a particular focus on total fat and saturated fatty acids, which are mandated for inclusion on labels in the European Union. The PBMS category demonstrated considerable diversity in formulation, particularly in terms of protein composition and fat sources (Table 1). Peas were identified as the primary protein source in most PBMS products (five products). According to the labels, three PBMS utilized a single type of oil as a fat source, whereas another three employed a combination of two oils. These products incorporated rapeseed, sunflower, and coconut oils. Such variations result in a broad spectrum of nutritional profiles compared to beef products. According to the detailed information on the label (Supplementary Table 1), PBMSs exhibited a highly diverse composition of ingredients, whereas BBPs constituted a much more uniform group. Consequently, PBMSs are diverse not only in their lipid composition but also in their energy content and macronutrients.

**Table 1:** Characteristics of the products included in the study based on the main protein and fat source.

**Sample ID**	**Product code**	**Main protein source**	**Main fat source**
1	A	soy, wheat	rapeseed oil, sunflower oil
2	B	pea	coconut oil
3	C	pea	rapeseed oil, coconut oil
4	D	wheat	n.d.
5	E	pea	sunflower oil
6	F	soy, pea, wheat, mushroom	sunflower oil
7	G	pea	rapeseed oil, coconut oil
8	H	beef	beef
9	I	beef	beef
10	J	beef	beef
11	K	beef	beef
12	L	beef	beef

*n.d.: no data; product codes A-G denote plant-based meat substitutes, H-L denote meat products.*

**Figure 1:**
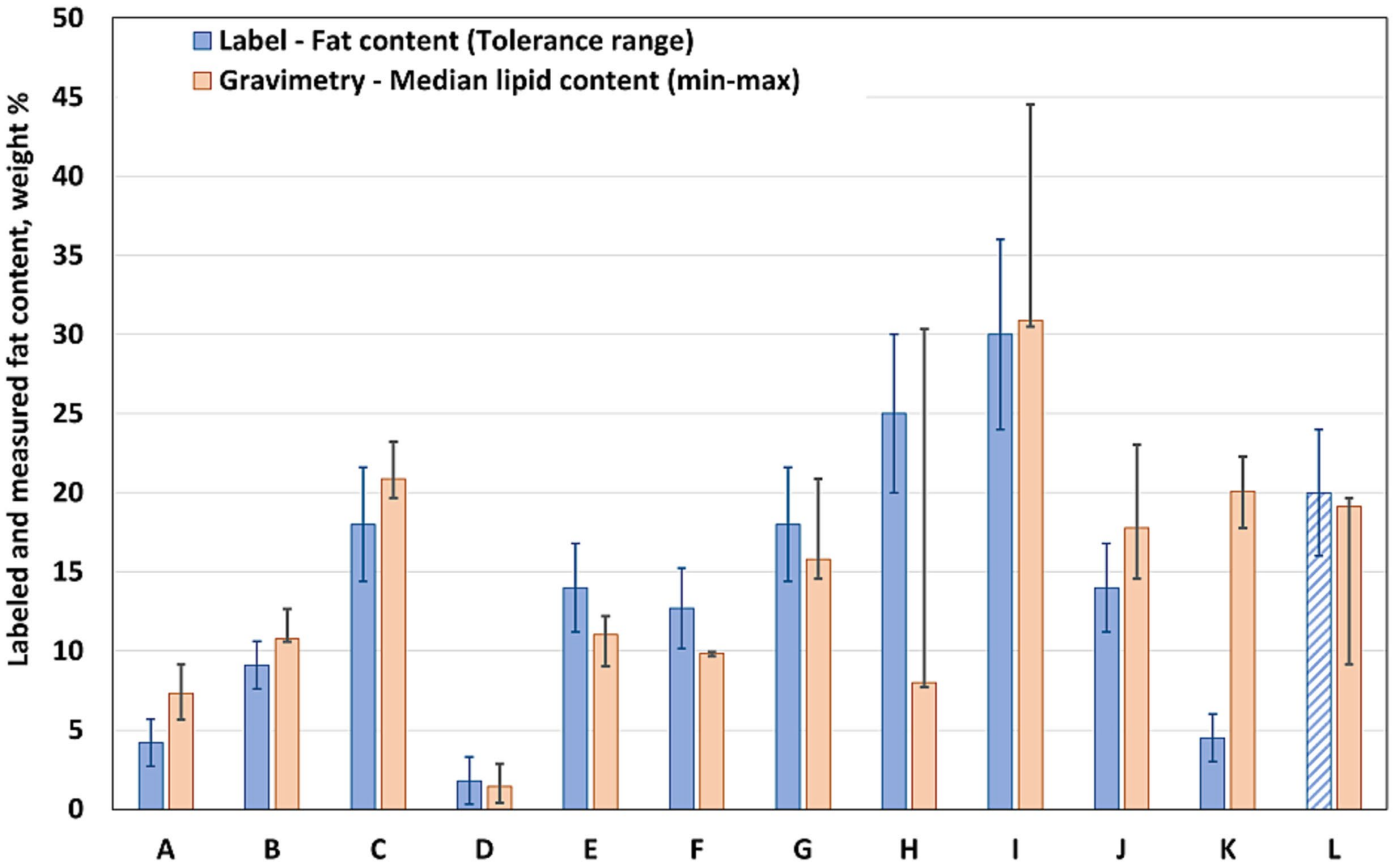
Comparison of total fat content on product labels (blue) with measured values (orange). Product codes A–G denote plant-based meat substitutes, and H–L denote beef-based products; the tolerance range for total fat is based on EU regulation (54).

We also assessed the concordance between the declared values and those obtained through chemical analysis. For each product, the total fat content indicated on the label was compared with laboratory measurements (Figure 1). SFA values were also examined when data were available (Table 2).

**Table 2:** Comparison of saturated fat content on product labels with measured values.

**Product code**	**Saturated fat content from label**	**Tolerance around the declared value of the saturated fat content**		**Saturated fat content from measurement**	**Deviation between declared and measured values**
	**(g/100 g)**	**Limits** (*: g/100 g)	**Range** **(g/100 g)**	**Median (Range)** **(g/100 g)**	**(%)**
A	0.5	±0.8*	0.0–1.3	0.80 (0.55–0.88)	+ 60
B	6.5	±20%	5.2–7.8	9.41 (9.16–11.01)	+ 45
C	5.4	±20%	4.3–6.5	7.15 (6.76–8.85)	+ 32
D	0.6	±0.8*	0.0–1.4	0.24 (0.07–0.50)	− 60
E	1.9	±0.8*	1.1–2.7	1.23 (0.94–1.34)	− 35
F	1.3	±0.8*	0.5–2.1	0.91 (0.85–0.93)	− 30
G	5.4	±20%	4.3–6.5	5.52 (4.79–8.02)	+ 2
H	10	±20%	8.0–12.0	3.70 (3.53–13.43)	− 63
I	16	±20%	12.8–19.2	17.41 (17.29–23.73)	+ 9
J	5.7	±20%	4.6–6.8	8.58 (7.07–11.17)	+ 50
K	1.9	±0.8*	1.1–2.7	9.56 (7.76–10.46)	+ 403
L	n.d.	n.d.	n.d.	8.50 (4.68–9.90)	-

n.d.: no data; *: total fat < 20 g / 100 g; product codes A-G denote plant-based meat substitutes, H-L denote beef-based products.

*Data are expressed as median (min, max) for measured total fat content (n = 3) and as median (min-max) for calculated saturated fat content (n = 12)*.

*The deviation between the declared and measured values was calculated based on the declared and median values of the measured saturated fat content*.

The declared total fat values in the products examined ranged from as low as 1.8 g/100 g in Product D (PBMS with no added oils) to 30 g/100 g in Product I (beef patty). Generally, PBMS products had a significantly lower median fat content (10.25% [8.60%; 14.87%], median [Q1; Q3]), with a maximum of 18 g/100 g, whereas BBPs had a higher median fat content (19.67% [16.16%; 26.68%]), reaching up to 30 g/100 g. The total fat content of PBMS varies widely, highlighting the diversity within this category. Although BBPs were more consistent in composition, with both protein and fat derived solely from beef and tallow, this apparent uniformity did not translate into similar total fat and SFA contents (Figure 1).

PBMS products containing coconut oil (Products B, C, and G) had higher SFA values than those formulated with only unsaturated oils (Products A, E, and F). In general, PBMSs had lower SFA content than BBPs; however, product K (a BBP) had a similar SFA content to PBMS products using unsaturated oils (Table 2).

In most samples, discrepancies were identified between the labeled values and analytical measurements (Figure 1). These inconsistencies varied in both magnitude and direction; approximately half of the products understated their actual fat content, whereas the other half overstated it. The deviations ranged from minor differences to substantial variations, exceeding 15 g/100 g. In a PBMS sample (Product A), the measured fat content was nearly double the labeled value. An even greater discrepancy was observed in a beef product (Product K), where the measured fat content was more than four times the declared amount, raising concerns regarding the reliability of the label claim. Conversely, overstatements were observed in some samples. A BBP (Product H) contained approximately 40% less measured total fat than that stated on the label.

Discrepancies were also observed between the declared and measured SFA contents (Table 2). As with the total fat content, products that underestimated the total fat content also tended to underestimate the SFA content. For most products, the measured values were 1.5 times higher than those indicated on the label. However, for one BBP (product K), the measured saturated fat content was five times higher than that indicated on its label. Conversely, another BBP (product H) contained only approximately 40% of the SFAs indicated on its label. The underestimated PBMSs contained 30–45% lower SFA than that stated on the label. One beef product (product L) did not contain quantitative fat content data, as this is not mandatory for minced meat products. The label only stated that the fat content was “not more than 20%.” According to laboratory analysis, the fat content was approximately 16 g/100 g, of which 8.5 g/100 g was saturated fat.

### 3.2 Fatty acid composition of the products

Table 3 presents the primary FA compositions for each PBMS (designated A–G) and BBP (designated H–L). The PBMS group exhibited considerable heterogeneity in FA profiles, reflecting their diverse lipid sources, whereas the BBP group demonstrated greater uniformity in FA profiles. Within the PBMSs, the predominant FAs were unsaturated oleic acid (C18:1n-9, OA) and LA, whereas in the BBPs, they were saturated palmitic acid (C16:0, PA) and monounsaturated OA. Lauric acid (C12:0) was nearly absent in most PBMSs (0–0.3 w/w% of total FAs), except in coconut oil-based products (Products B, C, and G). The product containing only coconut oil as the fat source exhibited the highest concentration of C12:0, exceeding half of the total FA content. In contrast, BBPs consistently displayed low C12:0 values.

**Table 3:** Main fatty acid composition of the plant-based and beef-based products.

w/w%	**Plant-based meat substitutes (PBMS)**							**Beef-based products (BBP)**					**PBMS vs. BBP**
	**A**	**B**	**C**	**D**	**E**	**F**	**G**	**H**	**I**	**J**	**K**	**L**	**p**
**C12:0**	^ABCD^ **0.05** [0.02]	^AEFGHIJKLM^ **55.23** [1.84]	^BENOPQRST^ **18.08** [3.63]	^FNUVab^ **0.06** [0.02]	^CGOUWXYZ12^ **0.27** [0.23]	**0.02** **[**0.01]	^DHVW34567^ **18.92** [5.38]	^IPaX3^ **0.09** [0.01]	^JQY4^ **0.07** [0.01]	^KRbZ5^ **0.08** [0.02]	^LS16^ **0.07** [0.02]	^MT27^ **0.08** [0.02]	**<0.05**
**C16:0**	^aABCDEFGHIJ^ **7.30** [0.93]	^aKLbMNOPQ^ **6.39** [0.59]	^AKRScTUVWX^ **5.27** [0.21]	^BLR^ **12.45** [0.44]	^CS^ **6.43** [0.23]	^Dc^ **5.87** [0.40]	^Eb^ **5.25** [0.48]	^FMT^ **25.87** [2.30]	^GNU^ **28.29** [1.49]	^HOV^ **25.17** [2.76]	^IPW^ **28.34** [2.77]	^JQX^ **28.57** [1.05]	**<0.001**
**C18:1n‐9**	^ABCDEa^ **59.18** [1.23]	^AFGHbIJ^ **6.71** [0.61]	^FKLab^ **43.77** [3.21]	^BKMNOc^ **24.55** [1.36]	^CLPdQ^ **28.49** [1.73]	^Daef^ **31.26** [0.15]	^GMPe^ **42.36** [3.47]	^HNd^ **41.12** [2.16]	^Eb^ **36.14** [3.00]	^ab^ **38.69** [1.62]	^IOQf^ **41.33** [3.22]	^Jc^ **38.43** [4.13]	**<0.05**
**C18:2n‐6**	^ABab^ **22.23** [0.75]	^Cc^ **3.80** [0.53]	^dD^ **13.77** [1.26]	^EFGH^ **55.10** [0.89]	^CIJKLM^ **59.18** [1.64]	^ceNOPQ^ **58.41** [0.12]	^fR^ **14.94** [1.45]	^Ie^ **3.72** [1.67]	^AdEJNf^ **1.15** [0.12]	^BDFKOR^ **0.73** [0.24]	^aGLP^ **1.51** [0.54]	^bHMQ^ **1.70** [0.15]	**<0.001**
**C18:3n‐3**	^AaBbCD^ **4.89** [0.28]	^cEd^ **0.75** [0.07]	^FeGfHI^ **4.98** [0.34]	^Jkghi^ **1.51** [0.17]	^jk^ **0.50** [0.10]	^AcFJL^ **0.19** [0.02]	^jLmMnNO^ **5.15** [0.43]	^am^ **0.50** [0.40]	^BEeGKkM^ **0.15** [0.03]	^Bfn^ **0.42** [0.13]	^CdHghN^ **0.22** [0.08]	^DIiO^ **0.26** [0.13]	**<0.001**

Each data point represents the median value of 12 measurements (three different expiration dates, two parallel chemical analyses, and two runs on a gas chromatograph for each sample).

Data are expressed as median [interquartile range].

Common superscript letters and numbers in the same row denote a significant difference between the given products: ^a,b,c^: 0.001 ≤ *p* < 0.05; ^A,B,C, 1,2,3^: *p* < 0.001.

The predominant SFA in beef, PA, was significantly more abundant in BBPs than in PBMSs (Table 3). In BBPs, the PA content remained within a narrow range, constituting approximately one-quarter of the total FAs. Conversely, in PBMSs, the PA content exhibited greater variability, ranging from 5 to 12.5%. The principal MUFA in BBPs was OA, which comprised approximately 40% of the total FAs. In contrast, PBMSs demonstrated substantial variability in OA content: one PBMS, formulated with high-OA oils (Product A), achieved nearly 60% of the total FAs, surpassing the BBP range, whereas the coconut-based PBMS (Product B) contained only approximately 6.5%, the lowest among all products.

The concentrations of the two EFAs (LA and ALA) were significantly lower in BBPs than in PBMSs (Table 3). The essential n-6 LA was found at low levels in BBPs, with a maximum of 3.7 w/w%, but was abundant in nearly all PBMSs. PBMS products enriched with sunflower oil (Products E and F) showed notably high LA levels of approximately 60%, whereas the product using only coconut oil (Product D) showed much lower values. The essential n-3 ALA levels were exceedingly low in BBPs (≤0.5 w/w%), whereas several PBMSs contained relatively high ALA levels. Formulations based on rapeseed oil (Products A, C, and G) contained approximately 5 w/w% ALA, which was approximately ten times higher than that in BBPs.

**Figure 2:**
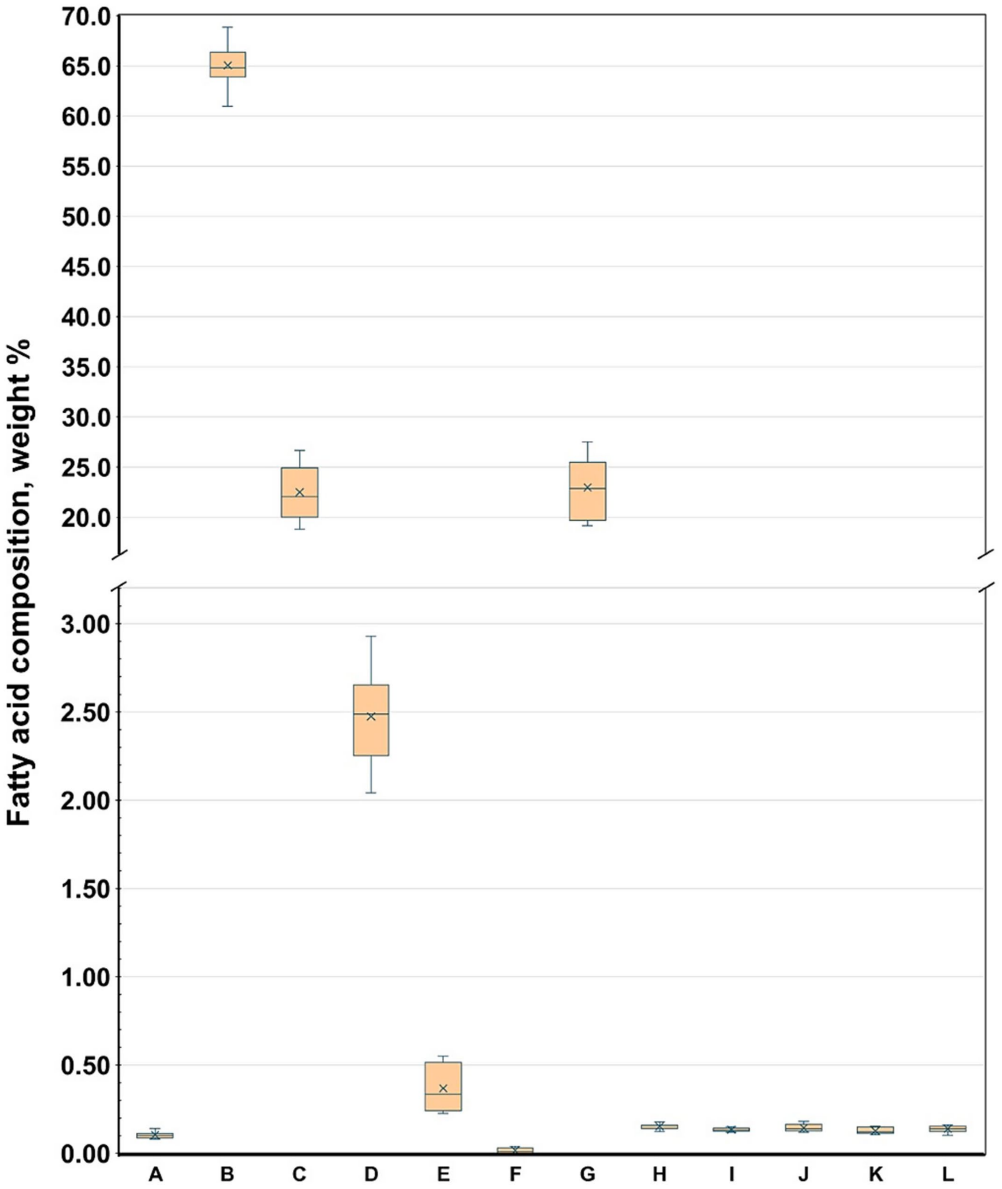
Sum of all medium-chain saturated fatty acids in the investigated products. Product codes A–G denote plant-based meat substitutes, and H–L denote beef-based products; sum of medium-chain saturated fatty acids denote: C6:0 + C8:0 + C9:0 + C10:0 + C11:0 + C12:0. Alt text: Box plot comparing the weight percentages of medium-chain saturated fatty acids across plant-based meat substitutes and beef products, showing that coconut-oil–based items, especially product B, have markedly higher levels, while all beef samples contain minimal amounts.

**Figure 3:**
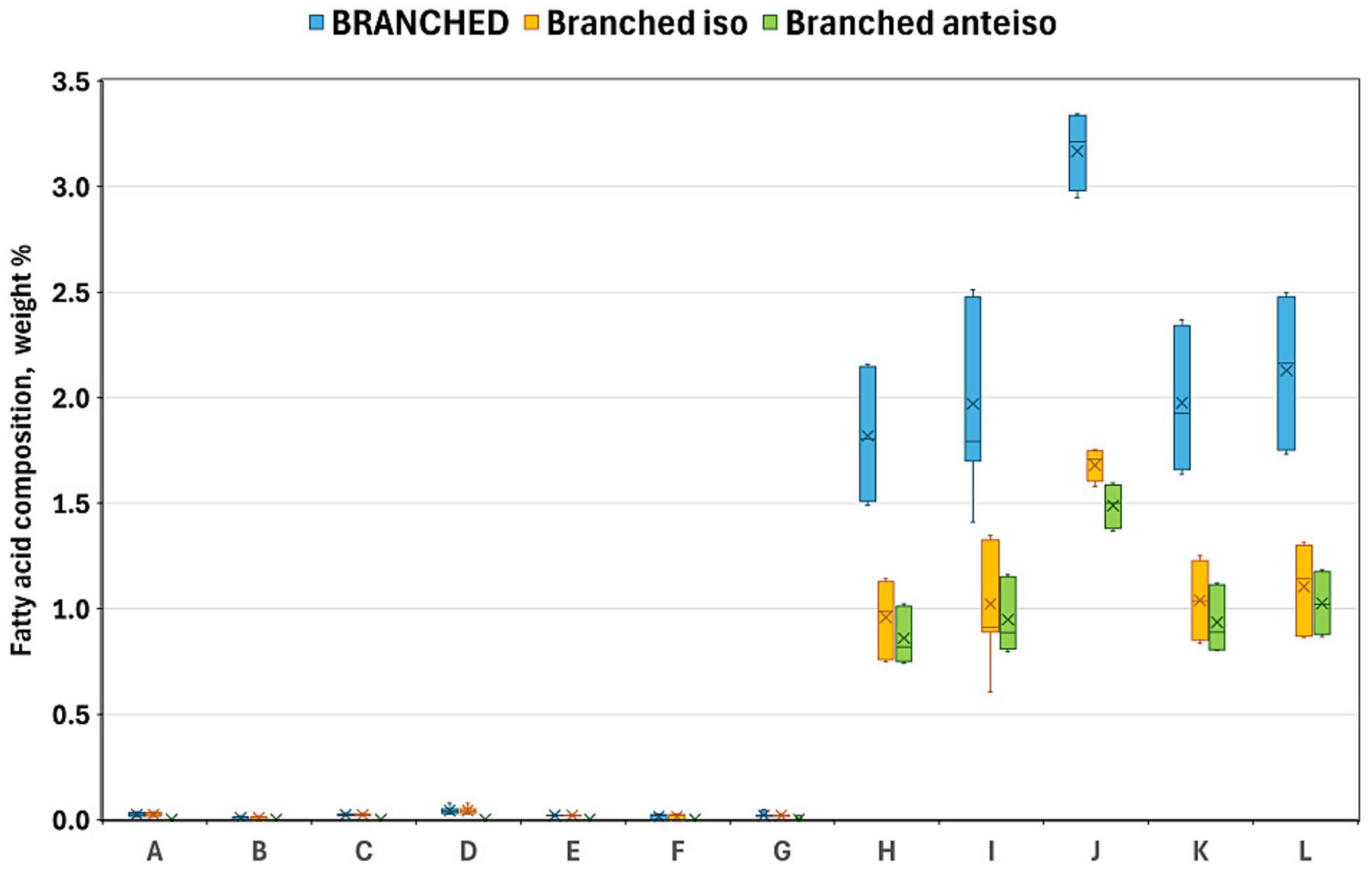
Sum of all branched-chain fatty acids (blue), iso (orange), and anteiso (green) branched-chain fatty acids in the investigated products. Product codes A–G denote plant-based meat substitutes, and H–L denote beef-based products; iso branched-chain fatty acids denote: C14: 0i + C15:0i + C16:0i + C17:0i + C18:0i; anteiso branched-chain fatty acids denote: C15:0ai + C17:0ai; all branched-chain fatty acids denote: sum of iso and anteiso branched-chain fatty acids. Alt text: Clustered box plot comparing total, iso-, and anteiso-branched chain fatty acid percentages in plant-based and beef products, showing that beef samples contain substantially higher levels (about 1–3% of total fatty acids), while plant-based alternatives contain almost none.

**Figure 4:**
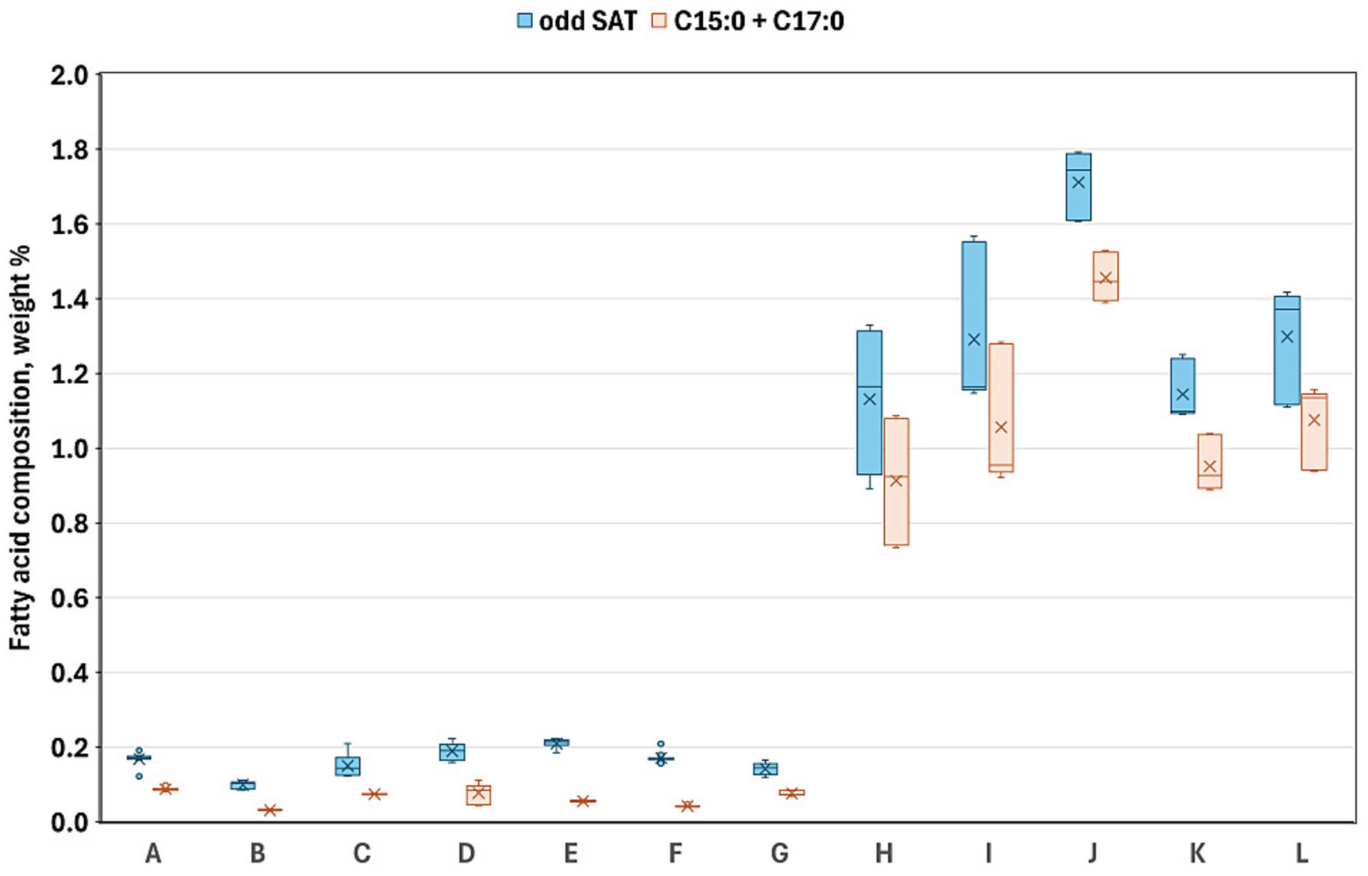
Sum of all odd-chain saturated fatty acids (blue) and sum of C15:0 + C17:0 (red) in the investigated products. Product codes A–G denote plant-based meat substitutes, and H–L denote beef-based products; sum of odd-chain saturated fatty acids denote: C9:0 + C11:0 + C13:0 + C15:0 + C17:0 + C19:0 + C21:0 + C23:0 + C25:0. Alt text: Clustered box plot comparing odd-chain saturated fatty acids, and sum of C15:0 and C17:0, between plant-based and beef products, showing markedly higher levels in beef samples (around 0.8–1.8%) and negligible amounts in plant-based ones.

Table 4 summarizes the fatty acid compositions of each product, highlighting the general compositional patterns. BBPs demonstrated significantly elevated levels of total SFA, MUFA, and *trans* isomeric fatty acids (TFA), while exhibiting reduced levels of n-6 and n-3 PUFAs compared to PBMSs. Consistent with the aforementioned individual fatty acids, the total SFA content in PBMSs exhibited considerable variability, ranging from approximately 10–85 w/w% of the total fatty acids. Notably, Product B, which exclusively utilized coconut oil as its fat source, was an outlier with an exceptionally high SFA content. In contrast, all BBPs consistently exhibited high SFA levels of approximately 50 w/w%. There were several significant differences in the SFA values, not only between the two groups but also among the products within each group. One group of SFAs, medium-chain fatty acids, was present in negligible amounts in BBP, whereas in the PBMS products containing coconut oil, this group constituted the majority of the total SFA (Figure 2). The highest value was observed in the product containing only coconut oil (Product B); however, in the other two products containing coconut oil along with other vegetable oils (Products C and G), values exceeding 20 w/w% were recorded, which is substantially higher than those found in the other products. In contrast, total branched-chain fatty acids, including both iso and anteiso branched-chain fatty acids, were present in BBPs at approximately 2 w/w%, whereas they were only found in very small amounts in PBMSs (Figure 3). Similarly, the concentrations of total odd-chain SFAs and the sum of the two major odd-chain SFAs, C15:0 and C17:0, were significantly higher in BBPs (above 1 w/w%) than in PBMS (Figure 4).

**Table 4:** Summarized fatty acid values of the plant-based and beef-based products.

w/w%	**Plant-based meat substitutes (PBMS)**							**Beef-based products (BBP)**					**PBMS vs. BBP**
	**A**	**B**	**C**	**D**	**E**	**F**	**G**	**H**	**I**	**J**	**K**	**L**	**p**
**SAT**	^AaBCbD^ **10.07** [0.98]	^AEFGHIJ^ **86.91** [1.33]	^EK^ **34.75** [5.21]	^FLcd^ **17.28** [0.45]	^GMNeO^ **11.01** [0.13]	^HPQRST^ **9.20** [0.49]	^If^ **34.96** [5.51]	^aJPf^ **45.75** [1.47]	^BKLMQ^ **56.14** [3.16]	^CcNR^ **48.31** [0.48]	^bdeS^ **47.11** [3.82]	^DOT^ **50.36** [6.24]	**<0.001**
**MUFA**	^ABCDEa^ **62.39** [1.47]	^AFGHaIJ^ **8.49** [0.81]	^FKb^ **46.35** [3.44]	^BKcLdMN^ **26.26** [1.44]	^CbOPe^ **29.28** [1.78]	^DfQ^ **32.01** [0.17]	^Gc^ **44.83** [3.63]	^HLOf^ **46.27** [4.98]	^Eg^ **39.38** [3.71]	^ad^ **43.62** [2.61]	^IMPQg^ **47.99** [2.94]	^JNe^ **43.79** [7.66]	**<0.001**
**TFA**	^ab^ **0.10** [0.05]	^aABCDE^ **0.01** [0.02]	^FGHIJ^ **0.03** [0.01]	^cKde^ **0.04** [0.01]	^fgLhi^ **0.04** [0.01]	^jMkm^ **0.04** [0.01]	^noNpq^ **0.04** [0.04]	^AFcfjn^ **1.05** [0.91]	^BGgo^ **0.90** [0.39]	^bCHKLMN^ **3.62** [1.48]	^DIdhkp^ **0.96** [0.49]	EJeimq **0.96** [0.46]	**<0.001**
**n-6 PUFA**	^ABab^ **22.28** [0.76]	^Cc^ **3.83** [0.53]	^dD^ **13.81** [1.31]	^EFGH^ **55.14** [0.89]	^CIJKLM^ **59.19** [1.64]	^ceNOPQ^ **58.44** [0.56]	^fR^ **14.98** [1.44]	^Ie^ **4.10** [1.80]	^AdEJNf^ **1.28** [0.15]	^BDFKOR^ **0.86** [0.27]	^aGLP^ **1.66** [0.54]	^bHMQ^ **2.05** [0.28]	**<0.001**
**n-3 PUFA**	^ABCD^ **4.89** [0.28]	^aEb^ **0.75** [0.07]	^cdeFgGH^ **5.02** [0.39]	^IJfg^ **1.51** [0.17]	^chi^ **0.50** [0.10]	^AadIK^ **0.19** [0.02]	^hKLjMO^ **5.15** [0.43]	^ei^ **0.64** [0.46]	^BEFJL^ **0.18** [0.05]	^gj^ **0.54** [0.17]	^CbGfM^ **0.26** [0.14]	^DHgO^ **0.34** [0.24]	**<0.001**

*Each data point represents the median value of 12 measurements (three different expiry dates, two parallel chemical analyses, and two runs on a gas chromatograph for each sample)*.

*Data are expressed as median [interquartile range]; Common superscript letters in the same row denote a significant difference between the given products: a,b,c: 0.001 ≤ p < 0.05; A, B, C: p < 0.001*.

*Abbreviations: SAT: sum of all saturated fatty acids, MUFA: sum of all cis monounsaturated fatty acids, TFA: sum of all trans isomeric fatty acids, n-6 PUFA: sum of all n-6 polyunsaturated fatty acids, n-3 PUFA: sum of all n-3 polyunsaturated fatty acids*.

Similar to the SFA values, the BBPs had a relatively uniform MUFA content (approximately 45%). In contrast, PBMSs ranged from very low MUFA (<10% in Product B) to very high MUFA (>60% in Product A) contents. PBMSs were essentially devoid of TFAs (<0.1%), whereas all BBPs contained significantly higher TFA levels (~1–3.5%). The differences in PUFA content were even more pronounced. Both n-6 and n-3 PUFAs were significantly higher in PBMSs than in BBPs. The n-6 PUFA values varied from approximately 3–4 w/w% to nearly 60% (Product E) in PBMS products formulated with seed oils. The total n-3 PUFA content was considerably higher in the PBMS group than in the BBPs, reflecting the inclusion of ALA-containing oils as a fat source. Several PBMS products contained approximately 5% n-3 PUFA (those with rapeseed oil), whereas all beef products contained very low levels of n-3 PUFAs. PBMSs presented a much broader and more heterogeneous FA profile than that of BBPs. In some PBMSs, depending on the oil used, SFAs were predominantly C12:0, with smaller amounts of C14:0 and C8:0 (Product B, C, and G). In contrast, other products primarily featured SFAs, such as C18:0 and C16:0 (Supplementary Table S2). We also observed several differences in the n-3 and n-6 PUFA compositions. Notably, Product B contained PUFAs with 22 carbon atoms, and we detected 0.47 w/w% C20:4n-6 in Product G, whereas other PBMSs mainly included only the two EFAs, LA and ALA. Conversely, the FA composition of BBP was considerably more uniform, with fewer significant differences among individual products (Supplementary Table S2).

### 3.3 Nutritional indices

#### 3.3.1 Essential fatty acid index

The EFA index values (Table 5), which reflect the relative abundance of EFAs, were substantially higher (approximately 10–20 times) in PBMS than in BBPs. The highest EFA scores were observed for products D, E, and F, which exceeded 55. Products A, C, and G also demonstrated significant EFA content, whereas product B was an outlier in the PBMS group, owing to its low EFA content. In contrast, the BBP group exhibited a narrow range of values, underscoring the markedly lower presence of EFAs in BBP.

**Table 5:** Nutritional indices of fatty acids in the plant-based and beef-based products.

**Median** [IQR]	**Plant-based meat substitutes (PBMS)**							**Beef-based products (BBP)**					**PBMS vs. BBP**
	**A**	**B**	**C**	**D**	**E**	**F**	**G**	**H**	**I**	**J**	**K**	**L**	**p**
**EFA**	^ABab^ **26.95** [0.52]	Cc **4.56** [0.60]	de **18.78** [1.61]	fDEFG **56.61** [1.07]	CHIJKL **59.75** [1.59]	cgMNOP **58.60** [0.13]	hQ **20.09** [1.88]	fHg **4.25** [2.05]	AdDIMh **1.32** [0.12]	BeEJNQ **1.15** [0.37]	aFKO **1.66** [0.52]	bGLP **2.00** [0.24]	**<0.001**
**n-3/n-6 PUFA**	aAB **0.22** [0.02]	bC **0.19** [0.01]	DEFcde **0.37** [0.01]	aDGH **0.03** [0.00]	AbEIJ **0.01** [0.00]	BCFKLfg **0.00** [0.00]	GIK **0.35** [0.01]	cM **0.15** [0.07]	dN **0.14** [0.05]	HJLMNhi **0.63** [0.02]	efh **0.13** [0.11]	gi **0.16** [0.10]	**0.147**
**UI**	ABa **121.74** [0.91]	ACDEFGb **18.49** [1.99]	Cc **89.34** [8.07]	DHIde **140.63** [1.07]	EfJKLM **149.37** [1.26]	FgNOPQ **149.54** [2.00]	Gh **90.23** [7.78]	bfg **56.87** [0.52]	BcHJNh **42.55** [3.76]	aIKO **47.55** [1.11]	dLP **53.01** [2.84]	eMQ **49.51** [5.93]	**<0.001**
**AI**	ABab **0.09** [0.01]	AcCDEde **8.65** [1.05]	cf **0.67** [0.16]	CFgh **0.16** [0.01]	DGHiJ **0.08** [0.00]	EfjkKLMN **0.07** [0.01]	dj **0.66** [0.17]	ek **0.70** [0.06]	BFGK **0.96** [0.13]	agHL **0.81** [0.07]	iM **0.77** [0.14]	bhJN **0.84** [0.10]	**<0.001**
**TI**	AaBCDEF **0.16** [0.02]	AGbHIQ **2.43** [0.21]	GJd **0.26** [0.04]	abe **0.32** [0.01]	KLfMQ **0.22** [0.00]	HgNOhP **0.19** [0.01]	IRi **0.26** [0.03]	Bg **1.62** [0.05]	CJeKNQR **2.60** [0.33]	DdLOi **1.88** [0.06]	Efh **1.77** [0.26]	FMP **2.02** [0.44]	**<0.001**
**hHI**	ABabC **11.55** [1.46]	ADEFGH **0.15** [0.02]	Dc **2.20** [0.50]	EIde **6.42** [0.23]	FfJKLM **12.79** [0.11]	GNOPQR **15.13** [1.23]	Hg **2.17** [0.57]	fN **1.59** [0.17]	BcIJOg **1.22** [0.14]	adKP **1.40** [0.14]	bLQ **1.41** [0.27]	CeMR **1.31** [0.14]	**<0.001**
**HFI**	ABaCDEF **4.87** [0.13]	AGHIJb **0.40** [0.05]	GKLc **2.91** [0.37]	HMNdeQ **3.53** [0.20]	IhR **1.90** [0.31]	BKMOiR **0.86** [0.06]	JOPj **2.95** [0.40]	abi **1.48** [0.10]	CLNhP **1.04** [0.09]	Dd **1.33** [0.02]	Ee **1.33** [0.20]	FcjQ **1.26** [0.14]	**<0.001**

*Abbreviations denote: EFA: essential fatty acids, n-3/n-6 PUFA: n-3/ n-6 polyunsaturated fatty acids, UI: unsaturation index, AI: atherogenicity index, TI: thrombogenicity index, hHI: hypocholesterolemic/hypercholesterolemic index, HFI: healthy fatty index*.

#### 3.3.2 The n-3/n-6 polyunsaturated fatty acids

There was considerable variation in n-3/n-6 PUFA values (Table 5) among the PBMSs, with levels ranging from very low to 0.35. In contrast, almost all BBPs exhibited similar values of approximately 0.15, except for product J, which had a value approximately four times greater. The PBMSs could be categorized into two subgroups: one with a relatively high n-3/n-6 PUFA ratio (products A, B, C, and G) and another with a very low ratio (products D, E, and F). Furthermore, no significant differences were observed between the PBMSs and BBPs.

#### 3.3.3 Unsaturation index

The UI (Table 5), which reflects the degree of unsaturation in the lipid profile, was significantly elevated in PBMSs compared to that in BBPs, with a notable group difference. This finding indicates a higher prevalence of unsaturated FAs in the plant-based groups. The PBMSs exhibited a broad range of UI values (18.49–149.54), with the majority of samples, including products A, D, E, and F, demonstrating values exceeding 120, indicating elevated levels of PUFAs. Conversely, the BBPs consistently presented lower UI values, averaging approximately 50, with product H exhibiting the highest value within this group.

#### 3.3.4 Atherogenicity index

In the PBMS group, the AI values (Table 5) were significantly lower than those in the BBP group. Most PBMSs (products A, D, E, and F) demonstrated a markedly reduced atherogenic potential relative to BBPs, with only two products (C and G) exhibiting AI levels comparable to those of BBPs. The sole exception was product B, which had an AI exceeding 8, surpassing the average observed in BBPs and indicating a lipid profile characterized by atherogenic SFAs. BBPs consistently exhibited higher IA values, ranging from 0.70 to 0.96.

#### 3.3.5 Thrombogenicity index

TI followed a pattern similar to that of AI (Table 5), with significantly lower values observed in the PBMS group than in the BBP group. In the PBMS group, the values mainly ranged from 0.16 to 0.32, except for product B, which exhibited the highest TI values, reaching nearly 2.5. In contrast, the BBP group showed TI values ranging from 1.63 (product H) to 2.62 (product I), with most values exceeding 1.7.

#### 3.3.6 Hypocholesterolaemic to hypercholesterolaemic index

The hHI (Table 5), which reflects the equilibrium between cholesterol-lowering and cholesterol-raising FAs, was markedly elevated in the PBMS group, indicating a more advantageous lipid profile. Products A, E, and F attained the highest values, each exceeding 11. Conversely, product B demonstrated a significantly low ratio of 0.15, diverging from the group’s mean. The BBP values were confined to a narrow range of 1.22–2.20, underscoring the consistently low hHI profile characteristic of beef fat.

#### 3.3.7 Healthy fatty index

The HFI was significantly higher in the PBMS group than that in the BBPs group. Again, HFI values showed a wide range in PBMSs, reaching the highest values (above 2.9) in products A, C, D, and G, while two products (products B and F) had even lower values than BBPs. In contrast, the HFI of all the BBPs was approximately 1.3.

## 4 Discussion

In the present study, the lipid content of PBMSs was significantly lower than that of BBPs, with several differences noted in FA composition and nutritional indices. The median lipid content in PBMSs was approximately half that of BBPs, with values of 10.25 and 19.67%, respectively, consistent with the existing literature. Previous studies have similarly reported significantly lower fat and saturated fat contents in plant-based burgers than in beef-based burgers across various regions, including Australia (7.2 ± 4.8 vs. 13.7 ± 7.8; *p* = 0.001 and 1.5 ± 1.6 vs. 6.2 ± 4.1; *p* = 0.005) (45), the UK (10.3 ± 5.0 vs. 15.0 ± 6.8; *p* < 0.001 and 1.7 ± 1.5 vs. 6.6 ± 2.7; p < 0.001) (46), Brazil (8.91 ± 6.63 vs. 16.88 ± 4.38; *p* = 0.048 and 3.20 ± 4.19 vs. 6.21 ± 1.38; *p* = 0.056) (47), South Africa (7.3 ± 5.9 vs. 13.7 ± 4.2 and 2.2 ± 3.2 vs. 6.0 ± 2.8) (48), Sweden (15 vs. 20 and 6 vs. 26) (49), Europe (9.13 ± 4.56 vs. 14.87 ± 5.18 and 1.54 ± 1.68 vs. 6.45 ± 2.42) (50), Spain (8.4 vs. 12.6 and 1.9 vs. 5.1) (51), Hong Kong (10.125 vs. 16.85; p < 0.001 and 2.3 vs. 5.83; p < 0.001) (52), and the USA (53). A review comparing different meat products and their plant-based substitutes also found that plant-based alternatives generally have lower total fat and saturated fat contents (9.17 vs. 20 and 1.6 vs. 12, respectively) (32).

Our research identified significant discrepancies between the reported and actual lipid content of the products. According to European Union regulations (54), the permissible tolerance for fat content on labels is ±1.5 g if the fat content is below 10 g per 100 g of food, and ±20% if it ranges from 10 to 40 g per 100 g. In the Hungarian PBMSs, we observed both lower and higher lipid contents than declared; however, these values remained within the acceptable tolerance range. In contrast, within the BBPs, one product (K) exhibited a measured fat content approximately four times higher than declared, while another product (H) contained 40% less fat than indicated on the label, both of which are unacceptable. These discrepancies may be attributed to the inadequate homogenization of the products, as suggested by the significant distance between quartiles. Additionally, they may have resulted from variations in production time, as we collected three different samples with three distinct expiration dates. A study conducted in Spain indicated that both the fat content and FA composition of meat products exhibit seasonal variability; however, this variability does not exceed 6% (55). Furthermore, fat content may vary depending on the methods used for its determination (56). It is essential to acknowledge that such inaccuracies may affect the calculation of energy and fat intakes in patients. Previous studies have also reported that the measured fat content is significantly higher than that indicated on the labels (57, 58). In Colombian whey products designed for sports nutrition, the labels indicated lower macronutrient content, including fat content, than the values measured (59). Conversely, another study identified that protein bars and protein puffs contained approximately 50% more fat than indicated, whereas other products, such as peanut butter and Greek yogurt, exhibited a fat content that was 15–25% lower than labeled (60).

In recent decades, the food industry has actively engaged in the development of meat alternatives, with researchers exploring various meat analogs. Although other options are available, plant-based substitutes are the most extensively studied and widely marketed (61, 62). These alternatives aim to replicate the taste, texture, and overall sensory experience of meat products while minimizing the environmental impact of livestock farming. They are made from various proteins, including soy, pea, and wheat, and different types of fats (63, 64).

In Hungarian PBMSs, the primary fat sources were coconut, rapeseed, and sunflower oils, similar to those found in Italian (37) and Belgian plant-based burgers (65). These three vegetable oils are the most commonly used for PBMSs worldwide. However, in some countries, other oils are also common, such as olive oil in Brazil (31, 47) and canola oil in Hong Kong (52), South Africa (48), Canada (40), and the USA (38). Soybean oil is used in certain products in Hong Kong (52) and South Africa (48), whereas cocoa butter is used in PBMSs in South Africa (48) and the USA (38).

The FA composition of PBMSs exhibited considerable variation, not only compared to BBPs but also among different PBMS products. In these plant-based products, the primary fat source determines the FA profile of the product. Coconut oil predominantly comprises medium-chain fatty acids, with lauric acid being the most abundant (46.64–48.03w/w%) (66, 67). Consequently, lauric acid had the highest value in the product where coconut oil served as the sole fat source (product B). In the two products where coconut oil was combined with other vegetable oils, the lauric acid levels were significantly lower than those in product B but were still markedly higher than those in products without coconut oil. Conversely, the principal FA in rapeseed oil is OA (56.8–66.6 w/w%), a MUFA (33, 68, 69). Therefore, OA is the predominant FA in PBMSs utilizing rapeseed oil. However, this oil is also a substantial source of n-3 EFA, ALA (7.8–10.0 w/w%), resulting in high ALA values in the three products containing rapeseed oil. The primary FA in sunflower oil is n-6 EFA, LA (20.36–69.56 w/w%) (33, 70, 71); thus, in the three PBMS products employing this oil as the main fat source, LA was found in the highest concentration. Our findings concur with previous studies that reported high lauric acid levels in plant-based burgers made with coconut oil in Brazilian (171.35–326.28 mg/g total fat; mean±SD) (31), Italian (median: 23.83 w/w%) (37), Canadian (13.15 ± 0.01 w/w%; mean±SD) (40) and US PBMSs (26.91 mg/g) (38). In contrast, the addition of sunflower oil or rapeseed/canola oil resulted in elevated LA values (15.14–17.82 w/w%) (38) and ALA levels (4.39–7.17 w/w%) (40),.

The FA composition of the BBPs was more consistent and aligned with the existing literature. The two primary FAs in beef are PA and OA, comprising approximately 25% and 35–40% of the total FAs, respectively (37, 40, 41, 72). Beef-based hamburger patties and minced meat are high in saturated fats (45–55% of total fat) and MUFAs (40–48% of total fats), but they contain low levels of PUFAs, as previously described (37, 38, 40).

Compared to PBMSs, all BBPs exhibited measurable TFA values, primarily ruminant *trans* isomers, along with branched-chain and odd-chain fatty acids, mainly C15:0 and C17:0. These FAs are produced by bacteria residing in the rumen of ruminants, such as beef (73–76), although the human microbiome can also generate branched-chain fatty acids (77), and fermented products are rich sources of these FAs (78). Some evidence indicates that TFAs derived from ruminants may offer health benefits, such as lowering the risk of type 2 diabetes (79) and eczema (80), although the findings remain inconsistent.

Various indices can be used to assess the health impacts of different foods based on their FA composition (43). The EFA index, which summarizes the LA and ALA content of food, indicated that PBMSs using sunflower oil as the primary fat source had the highest values. Vegetable oils, rich in essential LA and ALA, resulted in a significantly higher EFA index in nearly all PBMSs compared to beef, except for the product that used only coconut oil as the main fat source. The n-3/n-6 PUFA ratio was highest in the three products that used rapeseed oil as the primary fat source. In contrast, the main FA in sunflower oil is LA, an n-6 FA, resulting in an extremely low ratio in PBMSs made with sunflower oil. This indicates a high level of n-6 PUFAs compared with n-3 PUFAs. The AI represents the ratio of the sum of the main saturated (primarily proatherogenic) fatty acids to the main unsaturated (primarily antiatherogenic) fatty acids (81). The TI reflects the thrombogenic potential of foods containing pro-thrombogenic (mainly saturated) and anti-thrombogenic (MUFAs, n–3, and n–6 PUFAs) fatty acids in varying proportions (81). Nutritional indices of plant-based alternatives often suggest potential health benefits, with lower AI (0.11 vs. 0.64) and TI (0.26 vs. 1.87) than that of beef (31). However, PBMSs made with coconut oil can exhibit higher AI and TI values than those made with beef due to their high saturated fat content [AI: 5.43 ± 0.51 vs. 0.64; TI: 4.23 ± 0.32 vs. 1.87 (31) and AI:1.47 vs. 0.77 (37)]. Similarly, vegetable oils, except coconut oil, are rich in unsaturated fatty acids, resulting in a significantly higher UI in most PBMSs than in BBPs. Generally, the wide variation in formulations means that nutritional profiles can differ substantially between products. According to the current nutritional recommendations of the World Health Organization (WHO) and European Food Safety Authority (EFSA), SFA intake should be as low as possible, preferably no more than 10% of the total energy intake, and it is recommended to replace SFAs in the diet with PUFAs or plant-based MUFAs. The total fat intake should be 20–35% of the total energy intake, while there are no current recommendations for cis MUFA and PUFA intake (26, 82). Based on the calculated nutritional indices, the FA composition of the PBMSs included in this study, except those containing coconut oil, was much closer to the recommended FA intake than that of BBPs. Therefore, certain PBMSs may be more beneficial than traditional BBPs in terms of FA intake. In the early 2000s, two additional indices were introduced to evaluate the health effects of foods: the hHI, which describes the relationship between hypocholesterolemic fatty acids (mainly OA and PUFAs) and hypercholesterolemic fatty acids (mainly SFAs) (83) and the health-promoting index (HPI), which is the inverse of the AI (43).

In the present study, products rich in SFAs (BBP and PBMSs made with coconut oil) exhibited significantly lower hHI values than those using vegetable oils. Consequently, the nutritional indices of PBMSs (except those made solely with coconut oil) suggest a cardioprotective potential with reduced atherogenic and thrombogenic potential compared to traditional BBPs. A relatively new index, the HFI, considers the complexity of the FA composition of food in a more detailed manner than previous indices (44). This index aims to better differentiate between products with varying FA compositions by applying higher coefficients to the numerator for FAs with more beneficial effects and lower coefficients for the same FAs in the denominator than in the numerator. In the present study, PBMSs made with sunflower oil or rapeseed oil had the highest HFI values, indicating the potentially most beneficial effects of these products, whereas the product made solely with coconut oil (product B) had an even lower HFI value than BBPs in general.

In recent years, there has been a growing interest in consuming PBMSs instead of meat, as PBMSs can contribute to a sustainable diet with a lower environmental impact, especially when compared to the production of beef and other ruminant meats (84). Traditional plant-based diets, such as vegetarian or vegan diets, offer several benefits over omnivorous diets, including reduced red meat consumption. However, plant-based alternatives to traditional meat products differ from whole-food plant-based diets, which focus on minimally processed foods such as legumes, grains, vegetables, and fruits. Although most PBMSs have lower energy, total fat, and saturated fat contents than meat, their high added salt content may be concerning (39, 85). According to the NOVA classification, PBMSs are categorized as ultra-processed foods (35), and their potential health effects are uncertain. However, their composition can vary in terms of protein, fat, and sodium content, making the Nutri-Score or nutritional profiling potentially more reflective of their nutritional quality (86, 87). We observed significant variability in the fat content and primary fat sources among PBMSs, resulting in a wide range of FAs and nutritional indices. Currently, there is limited evidence on the long-term benefits or potential adverse effects of replacing meat with PBMS. In an eight-week randomized controlled trial, PBMS improved certain clinical parameters, such as diastolic blood pressure and fasting fructosamine concentration, but its consumption was not superior to that of meat (88). Shifting from an animal-based to a plant-based diet clearly benefits cardiometabolic health and reduces all-cause mortality (15). Additionally, a systematic review of seven randomized clinical studies indicated that incorporating PBMS into the diet lowers total cholesterol and LDL cholesterol (89).

We must also acknowledge that our study has both strengths and limitations. The primary strength of this study lies in its thorough examination of all commercially available plant-based burger patty alternatives in Hungary, which were compared with beef-based products. To account for variability among the different batches, we collected and analyzed each product with three different expiration dates. In addition to analyzing the label information, we conducted analytical measurements to assess the total fat content and FA compositions of these products. To our knowledge, this is the first study to compare a broad range of nutritional indices of lipids, including the latest HFI, rather than being limited to the AI and TI. However, this study has several limitations. While we attempted to reduce variability between productions by using three different expiration dates, it is crucial to highlight that over an extended period, significant differences may arise in both the lipid and FA compositions of products manufactured during various production periods. This could be attributed to factors such as variations in the FA composition of the oils and oil blends used, processing and homogenization methods, and other technological changes. We focused solely on fat content and FA composition, neglecting other macro- and micronutrients, such as proteins, carbohydrates, or minerals, despite previous studies indicating notable differences between plant-based and beef-based products. Our objective was to investigate the composition of beef burgers and their plant-based alternatives, excluding other meat products and their plant-based substitutes, such as sausages, minced meat, meatballs, and other meat burgers (e.g., those made from pork, chicken, or mixed meat). This study focused exclusively on the Hungarian market, which limits the generalizability of the findings to the broader European market. Furthermore, it is important to recognize that the small sample size, consisting of six plant-based and four beef-based products, also represents a limitation of our study.

## 5 Conclusion

In this study, we observed significant variability in the fatty acid composition of PBMSs, as the oils used directly influenced the fatty acid profiles of the products. While BBPs predominantly contain SFAs and MUFAs, most PBMSs are rich in PUFAs. PBMSs may offer more beneficial physiological effects owing to their lower average fat content and more favorable fatty acid composition. However, PBMSs cannot be regarded as a uniform group because of the diverse types of oils used in each product. Nonetheless, the health implications remain uncertain, underscoring the need for further randomized clinical studies to explore the effects of substituting meat products with plant-based alternatives on human health. Overall, PBMSs present a viable option for consumers aiming to reduce meat consumption; however, their nutritional quality largely depends on the specific ingredients, including the type of vegetable oil and processing methods employed.

## Data Availability

The original contributions presented in the study are included in the article/supplementary material, further inquiries can be directed to the corresponding authors.

## Glossary

AA: arachidonic acid
AI: atherogenicity index
ALA: alpha-linolenic acid
AUC: area under the curve
BBP: beef-based products
DHA: docosahexaenoic acid
EFA: essential fatty acid
EPA: eicosapentaenoic acid
FA: fatty acid
FID: flame ionization detector
GC: gas chromatography
HFI: healthy fatty index
hHI: hypocholesterolemic/hypercholesterolemic index
HPI: health-promoting index
IARC: International Agency for Research on Cancer
IQR: interquartile range
LA: linoleic acid
LDL: low-density lipoprotein
MUFA: monounsaturated fatty acid
OA: oleic acid
PA: palmitic acid
PBMS: plant-based meat substitute
PTV: programmed temperature vaporization
PUFA: polyunsaturated fatty acid
SFA: saturated fatty acid
TFA: *trans* isomeric fatty acid
TI: thrombogenicity index
UFA: unsaturated fatty acid
UI: unsaturation index
