# Supplementary material for: Fatty acid composition of ground-beef products and their plant-based meat substitutes available in Hungary
Source: Front Nutr. 2026 Feb 25;13:1732327. doi: 10.3389/fnut.2026.1732327 (PMC12975892; doi:10.3389/fnut.2026.1732327)
Supplement: Supplementary file 1 [file Supplementary_file_1.docx]

Supplementary Material Captions

## Fatty Acid Composition of Ground-Beef Products and Their Plant-Based Meat Substitutes available in Hungary

Viktor Koczka ^1,2^, Tamás Marosvölgyi ^3,^*, Zoltán Szabó ^4^, Timea Dergez ^3^ and Éva Szabó ^2,^*

| Academic Editor: Firstname Lastname  Received: date  Revised: date  Accepted: date  Published: date  **Citation:** To be added by editorial staff during production.  **Copyright:** © 2025 by the authors. Submitted for possible open access publication under the terms and conditions of the Creative Commons Attribution (CC BY) license (https://creativecommons.org/licenses/by/4.0/). |
| --- |

^1^ Doctoral School of Health Sciences, Faculty of Health Sciences, University of Pécs, Pécs, Hungary; [koczka.viktor@pte.hu](mailto:koczka.viktor@pte.hu) (V.K.)

^2^ Department of Biochemistry and Medical Chemistry, Medical School, University of Pécs, Pécs, Hungary; [szabo.eva.dr@pte.hu](mailto:szabo.eva.dr@pte.hu) (E.S.)

^3^ Institute of Bioanalysis, Medical School, University of Pécs, Pécs, Hungary; [marosvolgyi.tamas@pte.hu](mailto:marosvolgyi.tamas@pte.hu) (T.M.); [timea.dergez@aok.pte.hu](mailto:timea.dergez@aok.pte.hu) (T.D.)

^4^ Institute of Nutritional Sciences and Dietetics, Faculty of Health Sciences, University of Pécs, Pécs, Hungary; [zoltan.szabo@etk.pte.hu](mailto:zoltan.szabo@etk.pte.hu) (Z.S.)

***** Correspondence: [szabo.eva.dr@pte.hu](mailto:szabo.eva.dr@pte.hu); [marosvolgyi.tamas@pte.hu](mailto:marosvolgyi.tamas@pte.hu)

**Supplementary Table S1**: Composition of the products based on the label information. Product codes A-G denote plant-based meat substitutes, and H-L denote beef-based products.

**Supplementary Table S2:** Detailed fatty acid compositions of plant-based and beef-based products. Each data point represents the median value of 12 measurements (three different expiry dates, two parallel chemical analyses, and two runs on a gas chromatograph for each sample). Data are expressed as median, interquartile range (IQR). Product codes A-G denote plant-based meat substitutes, and H-L denote beef-based products.
